# Perspectives of the journal

**DOI:** 10.1590/S1980-57642009DN20300001

**Published:** 2008

**Authors:** Ricardo Nitrini

**Affiliations:** Editor-in-Chief

## First Meeting of the Advisory Editorial Board

In July 2008, during the International Conference on Alzheimer Disease held in Chicago, Illinois, the first meeting of the Advisory Editorial Board of **Dementia & Neuropsychologia** was held. In this meeting we discussed strategies to improve and increase the sourcing of manuscripts from different regions and countries, and to streamline the peer reviewing process. A few new procedures were suggested and approved, such as the establishment of junior editors to help the senior editors in the first reading of the manuscripts plus the inclusion of a checklist to be filled out by the author(s) before submitting the manuscript.

## Brazilian Association of Geriatric Neuropsychiatry

The most important and unanimously approved decision taken at the Advisory Editorial Board meeting was to elect **Dementia & Neuropsychologia** (currently the official journal of the Scientific Department of Cognitive Neurology and Ageing of the Brazilian Academy of Neurology) as the official journal of the Brazilian Association of Geriatric Neuropsychiatry (BAGNP). The BAGNP was founded in 1996, with the remit of disseminating knowledge to improve the quality of life in elderly impaired by neuropsychiatric disorders. The BAGNP brings together physicians from the three medical specialties whence it derives its name, as well as clinicians, neuropsychologists, physiotherapists, occupational therapists, social assistants, speech therapists and gerontologists, and has being pivotal in developing proficient professionals in this multidisciplinary area in Brazil. The BAGNP has fostered research and promoted the dissemination of results and conclusions of research through symposia and conferences held nationwide.

In this broadening of the readership of **Dementia & Neuropsychologia**, all members of BAGNP will be receiving the journal, as will members of the Scientific Department of Cognitive Neurology and Ageing of the Brazilian Academy of Neurology, allowing articles published in the journal to swiftly reach a large proportion of the professional and research community involved in the areas of interest of **Dementia & Neuropsychologia** in Brazil.

## Our jornal online

Another development to our readers and collaborators is that **Dementia & Neuropsychologia** is now available online. All articles published since its launch can now be easily accessed in pdf format on the Brazilian Academy of Neurology site (abneuro.org), via a convenient home page link. This valuable support by the Brazilian Academy of Neurology and its Scientific Department of Cognitive Neurology and Ageing is gratefully acknowledged.

## From abroad

Last but not least, in this issue of **Dementia & Neuropsychologia** we have the satisfaction of publishing papers from two Argentinian research centers. This a strong vote of confidence of these collaborators from a neighboring country in the potential of the journal as well as proof of their involvement in the endeavor to create a more open journal to expedite publication of high quality research produced by our respective countries.

**Ricardo Nitrini** Editor-in-Chief

